# Topological Metal of NaBi with Ultralow Lattice Thermal Conductivity and Electron-phonon Superconductivity

**DOI:** 10.1038/srep08446

**Published:** 2015-02-13

**Authors:** Ronghan Li, Xiyue Cheng, Qing Xie, Yan Sun, Dianzhong Li, Yiyi Li, Xing-Qiu Chen

**Affiliations:** 1 Shenyang National Laboratory for Materials Science, Institute of Metal Research, Chinese Academy of Sciences, Shenyang 110016, China

## Abstract

By means of first-principles and *ab initio* tight-binding calculations, we found that the compound of NaBi is a three-dimensional non-trivial topological metal. Its topological feature can be confirmed by the presence of band inversion, the derived effective Z_2_ invariant and the non-trivial surface states with the presence of Dirac cones. Interestingly, our calculations further demonstrated that NaBi exhibits the uniquely combined properties between the electron-phonon coupling superconductivity in nice agreement with recent experimental measurements and the obviously anisotropic but extremely low thermal conductivity. The spin-orbit coupling effects greatly affect those properties. NaBi may provide a rich platform to study the relationship among metal, topology, superconductivity and thermal conductivity.

Because the topological concept was successfully introduced into insulators, various insulators can be classified into topological trivial and non-trivial states^1^,^2^,^3^,^4^, in which topological insulators are highlighting an exciting scientific frontier of the topological electronic states. In analog of insulators, semimetals can also be classified from topological points as trivial semimetals and topological non-trivial semimetals (TSMs). Among TSMs, there are two classes of peculiar materials, topological Dirac semimetals (TDSs)^5^,^6^,^7^,^8^ and topological Weyl semimetals (TWSs)^9^,^10^,^11^,^12^,^13^,^14^,^15^,^16^,^17^,^18^, in which Fermi surfaces are consisted of isolated Fermi points in lattice momentum space. In general, the TDSs are predicted to exist at the critical phase transition point from a normal insulator and a topological one through the spin-orbit coupling effect or by tuning the chemical composition^19^,^20^. However, such bulk Dirac points are occasionally degeneracies and not stable. Interestingly, very recently the systems of the *P*6_3_/*mmc*-Na_3_Bi^6^,^7^,^21^,^22^ and *β*-BiO_2_^23^ and Cd_3_As_2_^8^,^24^,^25^,^26^,^27^ have been predicted theoretically and then Na_3_Bi and Cd_3_As_2_ have been experimentally confirmed to be robust TDSs protected by crystal symmetry. TWSs have been theoretically suggested to appear in skutterudite-structure pnictides^14^, pyrochlore iridates^16^, doped compound Hg_1−*x*−*y*_Cd*_x_*Mn*_y_*Te^15^ and some constructed heterostructures^17^, but to date no experimental verification has been achieved.

Certainly, there is no doubt that the topological concept can be also introduced into metals. Hence, metals would be also classified into two typical types of trivial metals (Ms) and non-trivial topological metals (TMs). In fact, many studies have been focused on the realization and the properties of TMs^28^,^29^,^30^,^31^,^32^,^33^,^34^,^35^,^36^,^37^,^38^. In general, the TMs can be achieved just by the effects of imperfections (*i.e.*, chemical doping, strain engineering, and heterostructure)^29^,^30^,^31^,^32^,^33^,^34^,^35^,^36^,^37^,^38^ and even by pressure^39^ and temperature (phonon)^40^ on topological insulators, insulators and semimetals. To date, several TMs were suggested to even occur at their native forms of *α*-Sn and HgTe^41^. It needs to be emphasized that TMs would indeed extensively exist. Nevertheless, because the topological non-trivial states of specified TMs' surface crossing the Fermi level can be easily mixed by trivial metallic bands, the real realization of TMs with the striking observable effects indeed poses a challenge.

Within this context, through first-principles calculations with the framework of Density Functional Theory (DFT) by employing the VASP code^42^,^43^, here we reported a native 3D TM, NaBi, which exhibits the combined interesting properties of the electron-phonon induced super-conductivity and the obviously anisotropic but extremely low bulk thermal conductivity. Its topological feature has been analyzed according to the band inversion occurrence between Na-*s* and Bi-*p* orbits at the Γ point, the Z_2_ number based on the derived parities, and the two selected surface non-trivial helical states. Without (with) the spin-orbit coupling (SOC) effect the super-conducting transition temperature of *T_c_* is derived to be 1.82–2.59 (2.92–3.75) Kelvin from the electron-phonon coupling strength *λ* = 0.71 (0.84) and the average velocity < *ω* >*_ln_* = 40.8 (38.7) cm^−1^, agreeing well with the experimental findings^44^,^45^. In addition, by considering phonon vibrational eigenvalues in the whole of Brillioun zone (BZ) and the phonon relaxation time derived from third-order force constants, we have further revealed that NaBi exhibits an extremely low lattice thermal conductivity but an obviously anisotropic feature of 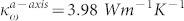 along the *a*-axis and 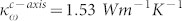 along the *c*-axis at room temperature, respectively.

As early as 1932, the compound of NaBi was synthesized to crystallize in a body-centered tetragonal CuAu-type structure (the space group of *P*4/*mmm*, No.123, see Fig. 1(a)) with Na at the 1*d* (1/2, 1/2, 1/2) site and Bi at the 1*a* (0, 0, 0) site^46^. The optimized DFT lattice constants^42^ of NaBi at the ground state, *a* = 3.4116 Å and *c* = 4.9530 Å, are in nice agreement with the experimental lattice constants^46^ (*a* = 3.46 Å and *c* = 4.80 Å). As illustrated in Fig. 1(c and d), NaBi is a typical metal. The SOC inclusion results in several apparent features. Without the SOC inclusion, the Fermi level lies in the declining shoulder of the densities of states (DOS), indicating a relatively high state of N(E*_F_*) = 0.85 states eV^−1^ f.u.^−1^. In contrast, the SOC inclusion significantly reduces the N(E*_F_*) to 0.52 states eV^−1^ f.u.^−1^, due to the fact that the Fermi level now stays at the valley of the pseudogap. In addition, from Fig. 1(d) in the occupied states of the DOS profile the SOC effect even induces the appearance of two obvious peaks dominated by Bi-*p*-like states at about −3 eV to −1 eV below the Fermi level, respectively. The presence of those features indicates the significance of the SOC effect for NaBi.

The SOC effect is even more obvious from the electronic band structures in Fig. 2(a and b). Firstly, without the SOC inclusion the three bands (as marked by No.1–3 in Fig. 2(a)) around the Fermi level heavily overlap each other along some high-symmetry lines. The large SOC effect results in their separations, as evidenced in Fig. 2(b). It interprets well as to why two main peaks (corresponding to No.1 and No.2 bands) occur in the occupied states of the DOS profile (Fig. 1(d)). In addition, due to the SOC separation between No.2 and No.3 the Fermi level now locates at the valley of the pesudogap. Secondly, from Fig. 2(a), because of the tetragonal symmetry with *c* > *a* and Bi atoms separated by the body-centered Na atom, at Γ the Bi *p_z_* orbital is lower in energy than both the degenerated Bi *p_x_*_,*y*_ orbital and the Na-*s* orbital. In particular, the band inversion and the anti-crossing feature between Na-*s* and Bi-*p_x_*_,*y*_ orbitals occur around Γ, showing a nontrivial gap of about 2.5 eV even without the SOC effect. It uncovers that this feature is indeed induced by both the crystal symmetry and the crystal field effect. Furthermore, under the SOC effect and the 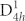 symmetry the doubly degenerated Bi-*p_x_*_,*y*_ orbitals are further split into 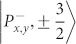 (as marked by *P*_1_ in Fig. 2(b)) and 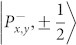 (as marked by *P*_2_ in Fig. 2b) states. This leads to a reduced non-trivial gap of about 1.1 eV between 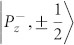 and 
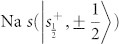
 states at Γ. Despite of the existence of the non-trivial gap, it is intrinsically different from topological insulators and topological semimetal since NaBi is a typical metal. Therefore, it would be extremely interesting to see whether or not NaBi is a non-trivial TM.

To answer this problem, the most important aspect is to elucidate whether or not the continuous energy gap exists between No.2 and No. 3 bands in the whole BZ in Fig. 2(b). On the one hand, we have performed the band structure calculations using a very dense *k*-mesh set (in total 187836 *k*-point number in the whole BZ, see Ref. 42) and the results demonstrated these two bands never touch each other at any *k*-point and, on the other hand, the calculations even uncovered that the smallest energy gap between No.2 and No.3 bands is about 0.08 eV at the four equivalent (
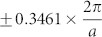
,
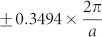
,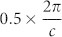) points in the *k*-space 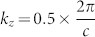 plane (here, *a* and *c* are the lattice constants)^42^. We have also constructed the tight-binding (TB) model Hamilton according to the DFT band structure with the SOC inclusion to further calculate the Berry phase of each energy band in the 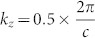 plane. The result uncovers that the Berry phase of the No.2 band is zero, thereby evidencing that the No.2 band never touches the No.1 and No.3 bands^42^. These results fully evidence the existence of a continuous gap between No.2 and No.3 bands in the whole BZ. Given the fact that the non-trivial gap exists between these two bands at Γ, we can further derive the topological invariant, according to the Berry curvature and connection^47^. Interestingly, for the center-symmetric structure (with the inversion symmetry) that NaBi crystallizes in, the effective Z_2_ invariant can be obtained in terms of the method proposed by Fu and Kane^41^. Because the bands below No.1 band are fully filled and far away in energy, the topological order just depends on the No.1 and No.2 bands starting from No.1 band around the Fermi level. As shown in Fig. 2(a and b), the product of the parities at the eight time-reversal invariant momentums (TRIMs) is −1, corresponding to *Z*_2_ of (1; 0 0 0). It indicates that NaBi is a strong 3D TM with the presence of the topological non-trivial states. It needs to be pointed out that the situation of NaBi is different from other known TMs of HgTe^41^, *α*-Sn^41^ and Bi_4_Se_3_^38^. For the latter, there are the intermediate states appearing within their inverted band gaps. For instance, for HgTe the doubly degenerated j = 3/2 states cross the Fermi level within the inverted band gap at the Γ point. The case of Sn is similar to that of HgTe. For Bi_4_Se_3_ in the inverted band Gap 2 as shown in Fig. 4 in Ref. 38 there exist two bands as the intermediate states. However, in the case of NaBi, there are no intermediate states in the inverted band gap induced by the crystal-field splitting.

We have further examined the intrinsic surface properties of NaBi. In principles, in similarity to topological insulators, TMs would have an odd number of Dirac cones to appear at any surface orientation because the topological order exists. However, for TMs the behaviors can be highly complex, mainly because the surface Dirac cones perhaps submerge into the bulk metallic states. Therefore, in some orientations it would have no chance to see the presence of surface Dirac cones for TMs. To prove these expectations, we shall now compute the band dispersions for the (001) and (100) surfaces using the *ab initio* TB model. The *ab initio* TB model is constructed by downfolding the bulk energy bands, obtained by first-principles calculations using maximally localized Wannier functions (MLWFs). The MLWFs are derived from atomic *p*-like and *s*-like states. The surface slab models (with the terminations of Bi atoms) for the (001) and (100) surfaces have been constructed with the thickness of 199 and 399 atomic layers, respectively. The results of the TB calculations are summarized in see Fig. 2(c and d). For the (001) surface, the surface electronic bands (as marked by the solid red circles) connecting the bulk electronic states derived from the No.2 and No.3 bands in Fig. 2(b) cross the Fermi level only once (odd number) for both 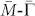 and 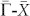. In addition, for this surface no Dirac cone appears because the surface electronic bands at 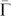 mix totally with the bulk electronic bands stemmed from the No. 3 band. However, the different behavior has been observed for the (100) surface [see Fig. 2b]. At 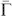 the clear Dirac cone appears with surface non-trivial states (as marked by solid red circles) which only once cut the Fermi level in the 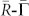 direction. In the 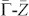 direction, there is no crossing at the Fermi level because the surface non-trivial states in this direction submerges into the bulk band states derived from the No.2 band. From the viewpoint of the topology, the cutting number of the Fermi level can be adjusted in different odd number just by shifting Fermi energy (such as chemical electronic and hole doping treatments). All these facts further evidence that NaBi is a 3D non-trivial TM.

We have utilized the linear response theory and fine *k* and *q* meshes^42^,^48^ to calculate the phonon dispersion, phonon density of states (PHDOS), Eliashberg function (*α*^2^F(*ω*)), and the strength of the electron-phonon (*e*-ph) coupling (*λ*(*ω*)) with and without the SOC inclusion. The phonon spectrum and the phonon densities of states in Fig. 3(a and b) can be divided into two main regions with mostly Bi (but also slightly mixed with Na) modes (0–60 cm^−1^ for SOC and 0–85 cm^−1^ for non-SOC) and highly pure Na modes (80–155 cm^−1^) for SOC and 85–170 cm^−1^ for non-SOC). As can be inferred for the phonon DOSs in Fig. 3(b), the SOC inclusion results in the average softening of over 15% for the transverse modes, and about 10% for the longitudinal ones. The Eliashberg function integrates to a large *e*-ph coupling strength *λ* = 0.72 (0.84) without (with) the SOC inclusion but gives the highly low logarithmic average < *ω* >*_ln_* = 40.9 (38.7 for SOC) cm^−1^. Although *λ* is very close to the value of *λ*~0.8 for MgB_2_ which mainly comes from high-frequency boron modes^51^, from Fig. 3(c) it is very clear that nearly over 95% of *λ* in NaBi is generated by the dominated Bi modes in the low-frequency acoustic branches. Strikingly, < *ω* >*_ln_* in NaBi is found to be only one tenth of the MgB_2_ value of ~ 450 cm^−1^^51^. Using the Allen-Dynes formula^52^ and typical *μ* of 0.14–0.10 we further estimate the *T_c_* in NaBi to be 1.82–2.59 K (2.92–3.75 K for SOC) (see Table 1). Although the estimated data without the SOC inclusion yields a perfect agreement with the experimental data^44^, the SOC inclusion indeed exhibits a significant effect on these superconducting parameters.

In particular, it needs to be emphasized that the compound of NaBi have two types of Fermi surfaces: one is a 2D hole Fermi surface (Fig. 1(b)) processing such a shape of quite tetragonal prism centered at the zone center Γ and the other one is a 3D electron Fermi surface (Fig. 1(b)) centered at the zone corner *A*. These two 2D and 3D Fermi surfaces are obviously originated from the No.2 and No.3 bands (see Fig. 2(b)), respectively. In addition, we also noted that, from Fig. 1(d) since the large gradient of the DOS around the Fermi level which just locates at the valley, the superconducting properties of NaBi may be highly affected by chemical impurities, vacancies, and external strains.

Furthermore, utilizing the frequency (*ω*) of phonon and the phonon group velocity (*v*) in the given transport direction and the derived relaxation time (*τ*) at wave vector *q* and polarization *j* within the framework of linear Boltzman's equation, we further derived the bulk lattice thermal conductivity as a function of temperatures as follows,


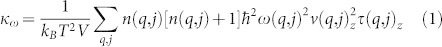
where *k_B_*, *V*, *n* and *z* are Boltzman constant, the crystal volume, and Bose-Einstein distribution as well as the direction of the thermal transportation. Specifically, the phonon's group velocity and specific heat per mode have been derived according to the second-order interatomic force constants obtained by the Phonopy code^49^. The relaxation time is in general determined by third-order force constants, which are derivatives of the total energy with respect to the atomic displacements in any three atoms *i*, *j*, and *k* in directions *a*, *b*, and *c* within a large supercell^53^. We have employed a real-space supercell approach to anharmonic third-order force constant calculations using the script of thirdorder.py^50^, which analyzes the symmetries of the crystal and significantly reduce the enormous number of DFT runs that would be required to characterize all relevant third-order derivatives of the energy. This method has been successfully applied to calculate the lattice thermal conductivity for a number of materials (such as, Si, diamond, InAs, and lonsdaleite, *etc*)^50^,^54^,^55^.

Currently, our derived temperature-dependent lattice thermal conductivities of NaBi have been compiled in Fig. 4. Remarkably, it can been seen that NaBi exhibits apparent anisotropic but extremely low lattice thermal conductivities. It has been also noted that the SOC effect plays an important role in affecting the *κ_ω_*. In comparison with the non-SOC case in Fig. 4, the SOC effect heavily reduced the thermal conductivities of both *a*- and *c*-axes directions. The mechanism is mainly attributed to the that the SOC inclusion indeed results in the softer phonon modes, as compared with those without the SOC effect (Fig. 3b). At room temperature, along the *a*-axis direction the *κ_ω_* is found to be 4.40 Wm^−1^K^−1^ (3.98 Wm^−1^K^−1^), whereas along the *c*-axis direction the *κ_ω_* is extremely low, only about 1.98 Wm^−1^K^−1^ (1.53 Wm^−1^K^−1^) with (without) the SOC effect. In particular, this low thermal conductivity is indeed comparable to those of widely known materials with ultralow thermal conductivities^56^,^57^,^58^,^59^, such as PbS, PbSe, PbTe, PtLaSb, and SnSe.

Interestingly, the low lattice thermal conductivity of NaBi exhibits an obviously anisotropic ratio of 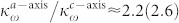 without (with) the SOC effect. Its anisotropy can be interpreted well, according to the group velocities of the phonon as illustrated in Fig. 4(b-m) in which the group velocities, *v_x_* along the *a*-axis (Fig. 4(a–f)) and *v_z_* along the *c*-axis (Fig. 4(b–m)), have been visualized as a function of the *k*-space distances between any phonon mode in the whole BZ and the zone centered Γ point. No matter whether the SOC effect is included, both *v_x_* and *v_z_* show the quite similar character, as evidenced in Fig. 4(b–m). The acoustic modes play a main role in determining the lattice thermal conductivities. In particular, along the *a*-axis direction the phonon group velocities are overall larger than those along the *c*-axis direction (Fig. 4(b–d) for a-axis and Fig. 4(h–j) for c-axis), thereby resulting in a higher *κ_ω_* along the *a*-axis. In general, the optical modes nearly makes no contributions to the *κ_ω_*. However, based on our calculations for NaBi, the optical modes make a certain contribution to the *κ_ω_* along the *a*-axis direction. For instance, at room temperature along the *a*-axis direction the *κ_ω_* that the optical modes contributed to is about 0.822 (0.742) Wm^−1^K^−1^ without (with) the SOC effect, being about 18% of the whole *κ_ω_*. This is mainly because its optical modes exhibit very large group velocities along the *a*-axis direction (Fig. 4(e–g)) and, in the meanwhile, the low frequencies (Fig. 3b) which are comparable to those of the acoustic modes. In contrast, from Fig. 4(k–m) the optical modes almost contribute nothing to the *κ_ω_* along the *c*-axis direction. This fact further enlarges the anisotropic ratio of the *κ_ω_*.

In summary, through first-principles calculations we have found that NaBi is an intrinsic 3D TM with the combined properties of electron-phonon induced superconducting and obviously anisotropic but extremely low lattice thermal conductivity. The SOC effect has been demonstrated to have the significant impacts on those properties. Compared to topological insulators and topological semimetals, our results for NaBi suggest that the topological metal can be realized in a simple body-centred tetragonal structure without any doping or strain treatments.

## Methods

For the standard DFT calculations, we have employed the VASP code^60^ with projector-augmented-wave (PAW) pseudopotentials and Perdew-Burke-Ernzerhof (PBE) exchange and correlation functionals^43^ by adopting the POTCARs of Na pv (PAW PBE Na pv 19Sep2006) and Bi d (PAW PBE Bi d 06Sep2000). The plane-wave energy cutoff was chosen as 350 eV and the k-mesh was set to 11 × 11 × 11 grid for the structural minimization. The optimized lattice constants are compiled in Table s1^42^. The lattice optimization, the electronic structures and Fermi surface of NaBi have been calculated with and without a fully relativistic spin-orbit coupling approach^61^,^62^.

Furthermore, the DFT calculations have been further performed with the PWSCF code of the Quantum Espresso package^48^. For the scalar-relativistic calculations (non-soc), we have used the normal-consuming potentials (Bi.pbe-mt-fhi.UPF and Na.pbe-n-mt bw.UPF recommended by QE). For the full-relativistic calculations (soc), we have employed the pseudopotentials (Bi.rel-pbe-dn-kjpaw.UPF and Na.rel-pbe-spn-kjpaw.UPF) in the projector augmented wave (PAW) framework in which the exchange correlation energy is evaluated using the Perdew-Burke-Ernzerhof (PBE) Generalized Gradient Approximation (GGA). The optimized lattice constants for NaBi have been further compiled in Table s1^42^. For all our calculations, the cutoff energies of 60 Ry and 500 Ry have been used for the wave functions and charge density, respectively.

The phonon dispersions with/without the SOC effect have been calculated within the linear-response theory using the QE Phonon code. A 24 × 24 × 16 k-point mesh with a Gaussian smearing of 0.05 Ry and a 4 × 4 × 4 q mesh are used for phonon dispersion calculations. For the electron-phonon coupling calculations, we have used the 24 × 24 × 16 k-point mesh^48^. In combination with VASP code, we have also derived phonon dispersions using the finite-displacement method to calculate the second-order force constant within the supercell (3 × 3 × 3 unit cells) by employing the code of Phonopy^49^. We have found that the derived phonon dispersions for NaBi with/without the OC effect are quite similar to those obtained by QE.

In addition, the second-order force constant is further applied to the calculation of the lattice thermal conductivity. In order to calculate lattice thermal conductivity, we have employed the recently developed code of ShengBTE^50^ in combination with ab initio code VASP. The main inputs are the sets of second- and third-order interatomic force constants. Currently, the second-order interatomic force constants can be calculated using the code of the Phonopy. The anharmonic third-order interatomic force constants can be calculated using the code of thirdorder.py^50^. In our calculations, it is based on a finite-difference 3 × 3 × 3 supercell approach, sharing a manner similar to Phonopy. In the first step, thirdorder.py generates a minimal set of displaced supercell configurations, then we can obtain the corresponding forces for these configurations using VASP code, and finally the script gathers the results to rebuild the whole anharmonic force constant set. In particular, a 3 × 3 × 3 supercell was employed for both second-order force constant calculations and the anharmonic ones. Only interactions up to third nearest neighbors were considered in the latter step for anharmonic case. In addition, the phonon's group velocity and specific heat per mode derived using the second-order interatomic force constants, and the relaxation time t derived from the third-order interatomic force constants, based on a full iterative solution to the Boltzmann transport equation.

## Author Contributions

X.-Q.C. conceived and initiated the study. R.H.L., X.Y.C., Q.X. and Y.S. performed the calculations. X.-Q.C., R.H.L. and X.Y.C. analyzed the calculated results. X.-Q.C. wrote the manuscript with helps from R.H.L. and X.Y.C.D.Z.L. and Y.Y.L. discussed the related calculations. All authors reviewed the paper.

## Supplementary Material

Supplementary Information Supplementary information

## Figures and Tables

**Figure 1:**
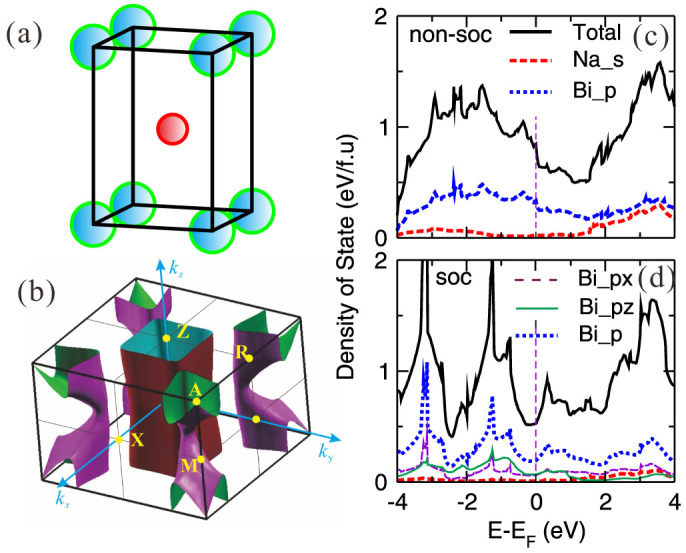
Structure, Fermi surface and densities of states (DOSs) of NaBi. (a) The unit cell, (b) the Fermi surface with the SOC effect, (c) and (d) the derived total and projected DOSs without and with the SOC effect, respectively.

**Figure 2:**
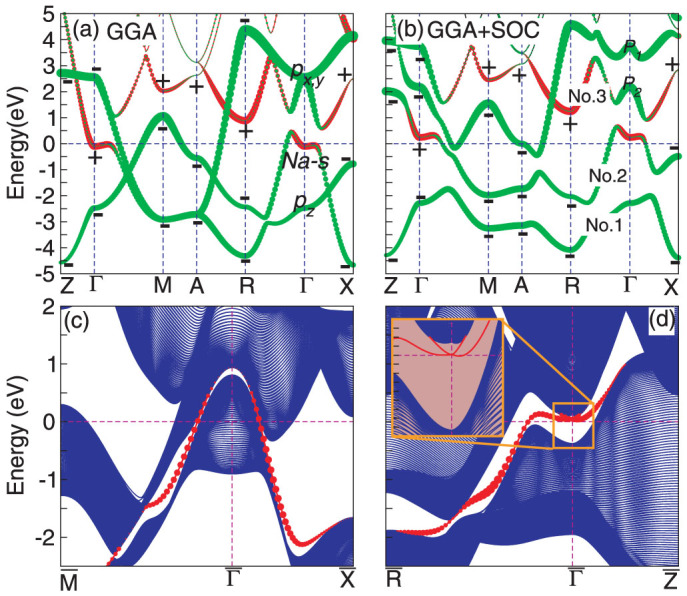
Bulk and surface electronic band structures of NaBi. (a) and (b) the DFT electronic band structures along the high-symmetry points without and with SOC, respectively. The signs “+” and “-” denote the parities of bands at the time-reversal invariant momenta (TRIMs). (c) and (d) corresponds to the (001) and (100) surface electronic band structures derived from the tight-binding model (supplementary materials). The red dots show the helical spin-resolved metallic states on the surfaces. Inset of the panel (d) displays the surface Dirac cone with the enlarged scale around the Fermi level at the Γ point.

**Figure 3:**
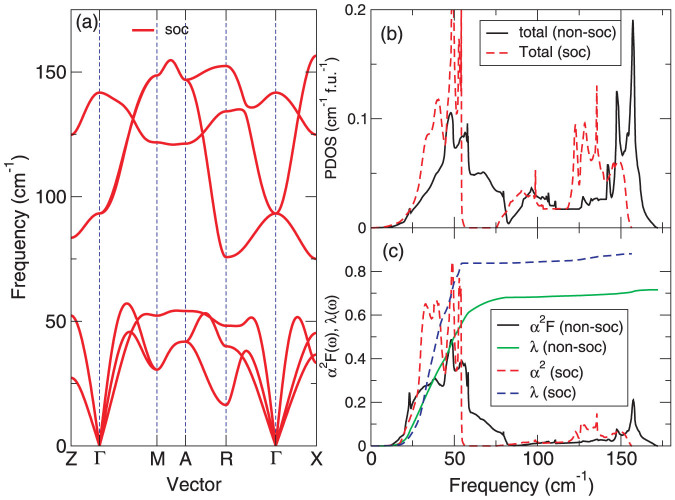
Phonon dispersion and electron-phonon coupling strength of NaBi with the SOC inclusion. (a) Phonon dispersion curves along the high symmetry lines of the BZ with the SOC inclusion, (b) total and projected phonon density of states (PHDOS) in NaBi with/without the SOC inclusion, (c) Eliashberg function and the strength of the electron-phonon coupling with/without the SOC inclusion.

**Figure 4:**
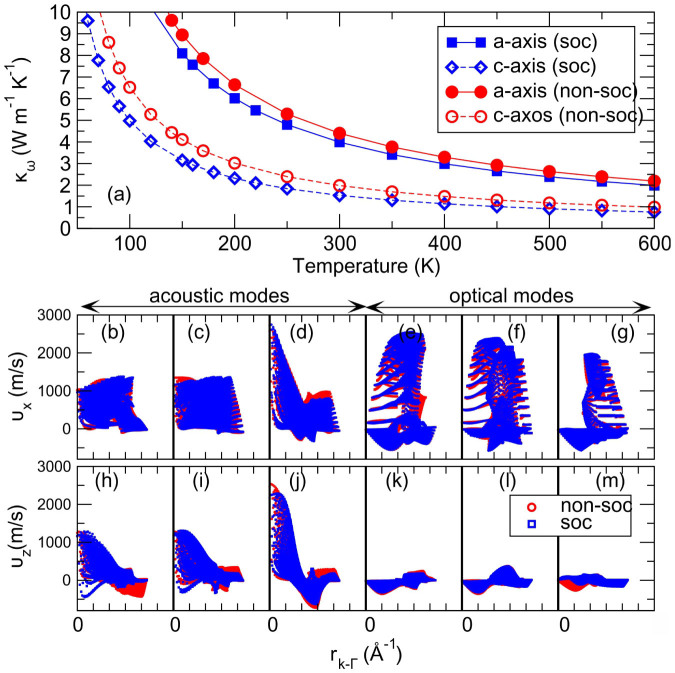
Upper panel: the derived lattice thermal conductivities along the *a*- and *c*-axis with and without the SOC effect, respectively. Lower panel: the phonon group velocities along the *a* and *c*-axis per phonon mode as a function of the distances between the given *k* point and the centered Γ point (in total, 125 000 *k*-point number in the BZ) with and without the SOC effect.

**Table 1:** Superconducting parameters (*λ*-electron-phonon coupling strength, <*ω* >*_ln_*-logarithmic average in cm^−1^, *T_c_*-superconducting transition temperature in *K*, and Θ*_D_*-Debye temperature in *K*) of NaBi without and with the SOC inclusion. Note that Debye temperature has been derived according to the elastic constants of NaBi with/without the SOC inclusion

	*λ*	< *ω* >*_ln_*	*T_c_*	Θ*_D_*
non-SOC	0.72	40.9	1.82–2.59	147.4
SOC	0.84	38.7	2.92–3.75	151.2
Expt Ref. 44	0.62		2.15	140.0
