# Supplementary material for: Topological Metal of NaBi with Ultralow Lattice Thermal Conductivity and Electron-phonon Superconductivity
Source: Sci Rep. 2015 Feb 13;5:8446. doi: 10.1038/srep08446 (PMC4327411; doi:10.1038/srep08446)
Supplement: Supplementary Information [file srep08446-s1.pdf]

Supplemental Materials on

Topological Metal of NaBi with Ultralow Lattice Thermal Conductivity and Electron-phonon Superconductivity

*Ronghan Li, Xiyue Cheng, Qing Xie, Yan Sun, Dianzhong Li, Yiyi Li, Xing-Qiu Chen**

Shenyang National Laboratory for Materials Science, Institute of Metal Research, Chinese Academy of Sciences, Shenyang 110016, China

(*corresponding author: xingqiu.chen@imr.ac.cn)

Contents

1 Band gap of NaBi between No.2 and No.3 bands

2 Calculations of Berry phase of No.2 band

3 Optimized lattice constants of NaBi

1 Band gap of NaBi between No.2 and No.3 bands

To elucidate whether or not the continuous band gap exists between No.2 and No.3 bands in the whole BZ of NaBi, we have performed a detailed analysis via four steps. According to the symmetry of NaBi, we only need to analyze the corresponding band gaps in the one eighth part [k_x(0,1/2), k_y(0,1/2), k_z(0,1/2)] of the whole BZ as illustrated in Fig. s1(c) (marked by thick lines).

In the first, we calculated electronic energy bands using a dense *k*-mesh (46×46×46 = 94336 *k* points) in the one eighth part [k_x(0,1/2), k_y(0,1/2), k_z(0,1/2)] of the whole BZ. Figure s1 lists all band gaps between No.2 and No.3 bands as a function of the distance between any *k* point among those 94336 *k*-points and the centered Γ point. In order to clearly visualize the specified position of the smallest band gap, we further plot the three-dimensional (3D) band gap evolutions (Fig. s2(a)) and its two-dimensional (2D) projection (Fig. s2(b)) as a function of the *k*-space position of the k_z=1/2 plane.

In the second, we calculated electronic energy bands in a smaller region [k_x(1/3,0.4), k_y(1/3,0.4), k_z(0.4,1/2)] using the very dense *k*-mesh (40×40×40 = 64000 k points), as illustrated in Fig. 1s. In the third, we will reduce the scale of the k-space in the second step into a smaller one [k_x(1/3,0.38), k_y(1/3,0.38), k_z(0.4792,1/2)]] using the very dense *k*-mesh (30×30×30 = 27000 k points), as illustrated in Fig. 1s. Finally, we perform a final dense calculations with a tiny k-space [k_x(0.34,0.35), k_y(0.34,0.35), k_z =1/2] using the highly dense k-mesh (50×50×1 = 2500 k points), as shown in Fig. 1s. The evolution of the band gap between No.2 and No.3 bands are visually in Fig. s2(d) by varying the k-space positions.

From the above four steps, all these calculations commonly revealed the existence of the continuous band gap between No.2 and No.3 bands at any k point in the whole BZ. The smallest band gap between No.2 and No.3 bands is about 0.08 eV at the (0.3461, 0.3494, 0.5) *k* point. According to the symmetry of NaBi, it can be further referred that in the whole BZ there should are four equivalent points (±0.3461, ±0.3484, 0.5) at which the band gap between No.2 and No.3 bands has the smallest value of 0.08 eV. Furthermore, we also plot the electronic band structure along the Z-(0.3461,0.3494,0.5)-A line (here Z and A are high-symmetric point of (0, 0, 0.5) and (0.5, 0.5, 0.5), respectively (see Fig. 2s(e)). From this figure, it can be clearly seen that the smallest band gap is about 0.08 eV at the (0.3461, 0.3484, 0.5) position, which has a distance of 0.1759 $Å^{-1}$ away from the Γ point.

2 Analyzing Berry phase of No.2 band

We calculated the band gap using the very dense k-point in the whole of BZ region and we found that the minimum band gap between No.2 and No.3 should be 0.08 eV at four *k* points in the $k_z=\frac{\pi}{c}$ plane. Although we used the much denser k-point number to doubly check the band gap between No.2 and No.3 in the $k_z=\frac{\pi}{c}$ plane, from the viewpoint of the numerical calculations it is still a bit

difficult to 100% guarantee whether or not we have found the k-point that exhibits the minimum band gap. Furthermore, we want to use the zero Berry phase to clarify further the fact that No.1 and No.3 bands ever touch with No2 band.

Firstly, we have defined a mirror (001) plane and its corresponding plane in the ***k*** space satisfies $\delta_{\vec{k}-\vec{k}',\vec{G}}$ in which $k' = \hat{M}(0,0,1)$, $\vec{k} = (k_x, k_y, -k_z)$ and $\vec{G} = n_1\vec{B}_1 + n_2\vec{B}_2 + n_3\vec{B}_3$ ($n_{i=1,2,3}$ is integer and $\vec{B}_{i=1,2,3}$ is the basis vector in the ***k*** space). Of course, the planes of （$k_x, k_y, k_z = 0$） and（$k_x, k_y, k_z = \frac{\pi}{c}$） satisfy the mirror symmetry with respect to the (001) plane.

Secondly, through the first-principles calculations using very dense k-point we have found that the minimum band gap appears at the k_z=$\frac{\pi}{c}$ plane. According to the mirror symmetry, we can define the eigenstate $|u_n^m(k_x, k_y, k_z = \frac{\pi}{c})>$ for both Hamiltonian $\hat{H}$ and the mirror operator $\hat{M}(0,0,1)$ at the k_z=$\frac{\pi}{c}$ plane. Note that here *n* corresponds to the band order without the consideration of the spin double degeneration, and $m = \pm i$ is the eigenvalue for the mirror symmetry. For each band *n*, $|u_n^{m=\pm i}(k_x, k_y, k_z = \frac{\pi}{c})>$ are doubly degenerated.

Thirdly, we can further calculate the Berry phase for the No.2 band in the k_z=$\frac{\pi}{c}$ plane through the formula as follows,

$$\gamma_n = i\oint < u_n^m(\vec{k}) | \nabla | u_n^m(\vec{k}) > d\vec{k} \qquad (1)$$

Note that here we only consider if there is any band crossing between No.2 and No.3 (without spin degeneration). For sake of the convenience, we calculated the Berry phase for *n*=2 and *m*=i, namely,

$$\gamma_n = i\oint < u_{n=2}^{m=i}(\vec{k}) | \nabla | u_{n=2}^{m=i}(\vec{k}) > d\vec{k} \qquad (2)$$

On the one hand, if there is not any band inversion or the band crossing between No.2 and other No.1 and No.2 bands, after the movement of one whole circle the eigenstate of $|u_2^i(k_x, k_y, k_z = \frac{\pi}{c})>$ will go back to itself and the eigenstate will achieve a geometry phase (Berry phase) of $2\pi N$, where $N(=0,1,2...)$ is an integer. On the other hand, if there is a band crossing between No. 2 and other No.1 (or No3) bands, the eigenstate should go into the other bands. When the eigenstate passes this crossing, the eigenstate does not go back to itself after one circle. With this situation, the calculated Berry phase by Equ. (2) does not equal to $2\pi N$, anymore. Therefore, in this case of NaBi we can use the Berry phase of No.2 as a criterion to identify the existence of band gap between No.2 and No.3.

3 Optimized lattice constants of NaBi

Table s1, the optimized lattice constants of NaBi at the ground state using by both VASP and QE codes.

	DFT	*a* (Å)	*c* (Å)
VASP	non-soc	3.4116	4.9530
VASP	soc	3.4613	4.8912
QE	non-soc	3.4534	4.9364
QE	soc	3.4874	4.9006
EXPT [1]		3.46	4.80

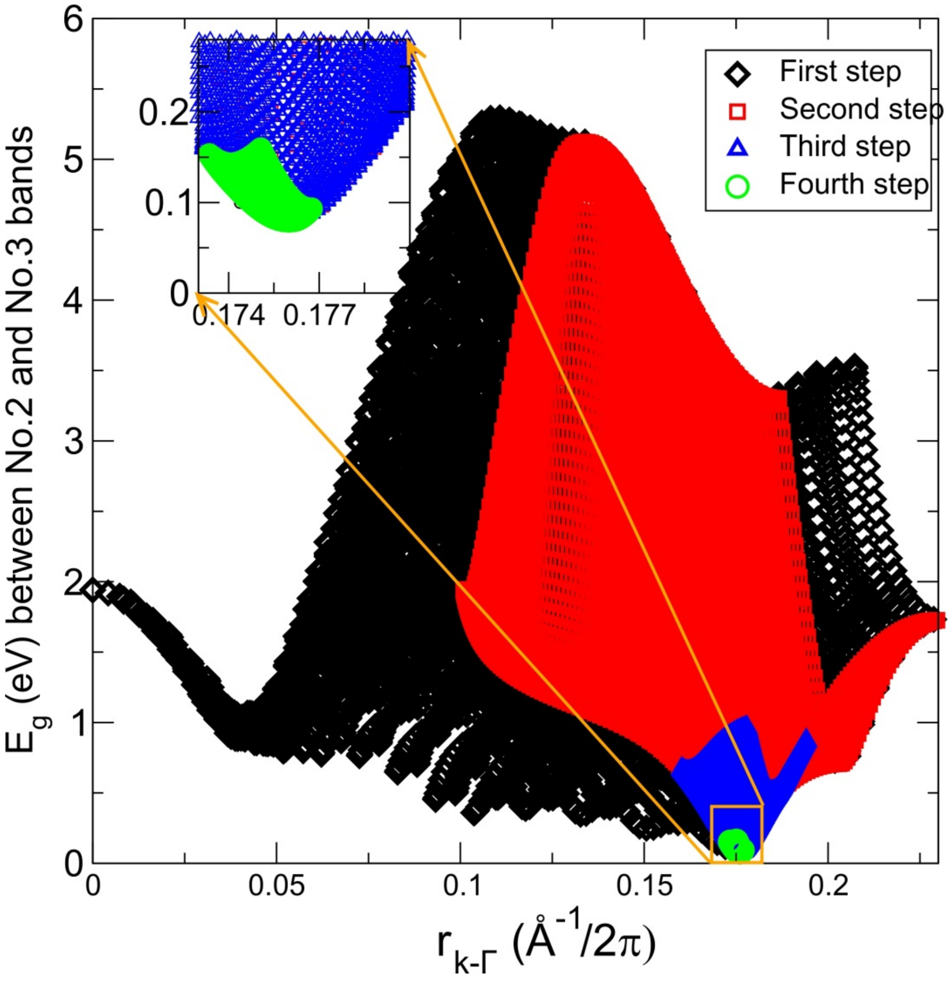

Figure s1. The calculated band gap between No.2 and No.3 bands of NaBi in the one eighth part of the whole BZ as a function of the distance between any *k* point and the zone centered Γ point. It can be seen that the smallest band gap is about 0.08 eV at the distance of 0.1759 Å^{-1} away from the Γ point.

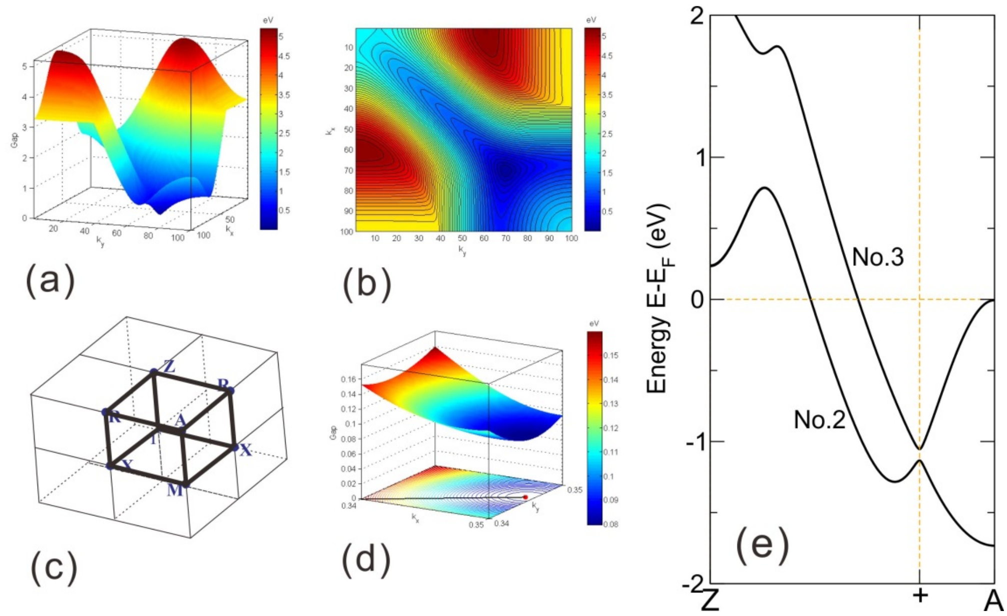

Figure s2. (a) and (b): the three-dimensional (3D) band gap evolutions and its two-dimensional (2D) projection in the k-space position for the k_z=1/2 plane, respectively. (c): the BZ region in which the thick lines highlight the one eighth part. (d): the three-dimensional (3D) band gap evolutions and its two-dimensional (2D) projection for the k_z=1/2 plane, respectively, in a very small area determined by [k_x(0.34,0.35), k_y(0.34,0.35), k_z =1/2].
